# Prevention strategies against academic burnout: the perspective of Romanian health sciences students in the aftermath of the COVID-19 pandemic

**DOI:** 10.3389/fpsyg.2025.1465807

**Published:** 2025-04-02

**Authors:** Ovidiu Popa-Velea, Ioana Ruxandra Stoian-Bǎlǎşoiu, Alexandra Mihai, Alexandra Ioana Mihǎilescu, Liliana Veronica Diaconescu

**Affiliations:** ^1^Department of Medical Psychology, Faculty of Medicine, “Carol Davila” University of Medicine and Pharmacy, Bucharest, Romania; ^2^Department of Psychiatry, “Prof. Dr. Alexandru Obregia” Clinical Hospital of Psychiatry, Bucharest, Romania; ^3^Department of Diabetes, Nutrition and Metabolic Diseases, “Elias” Emergency University Hospital, Bucharest, Romania

**Keywords:** medical, students, effective, prevention, burnout

## Abstract

**Background:**

Burnout in academia can have important long-term consequences. This study aimed to investigate academic burnout and the perception of its prevention strategies among Romanian health sciences students.

**Methods:**

Three hundred and five health sciences students (from General Medicine, Dental Medicine, Pharmacy, and Midwifery and Nursing) (36 men, 269 women, mean age 21.9, standard deviation 1.911) self-rated their burnout using the Burnout Assessment Tool (BAT) and a visual analog scale, and answered a survey with open and closed questions about the most effective coping strategies to prevent burnout.

**Results:**

Above average scores (mean = 3.05; SD = 0.67) on burnout were met (with the highest scores on exhaustion and psychological distress and the lowest on mental distance and psychosomatic symptoms). Women were more affected than men by burnout and associated emotional impairment and secondary symptoms. Participants rated supportive relationships as the most effective in preventing burnout (mean = 3.75; SD = 0.55), followed by relaxation-meditation (mean = 3.32; SD = 0.85), while they also positively valued cognitive-behavioral therapy (those with high burnout scores), and physical activity (those with low burnout scores). Organizational strategies have included re-evaluating the university curriculum (mean = 3.83; SD = 0.48) and increasing access to psychological support programs (mean = 3.72; SD = 0.58) and to programs that increase self-efficacy and resilience (mean = 3.65; SD = 0.61). The qualitative analysis showed that freely chosen individual strategies included, in descending order, detachment, active actions, healthy lifestyle, and specialist help, while the most preferred organizational were better academic organization and program optimization.

**Conclusion:**

These results may be useful in designing more sustainable and effective burnout prevention strategies in health sciences academic settings.

## 1 Introduction

Burnout syndrome is a continuously developing concept since its introduction in the 1970s, this being consistent with the increasing number of individuals suffering from exhaustion, regardless of their professional field (Heinemann and Heinemann, 2017; Calitz, 2022). According to the most recent description of the World Health Organization (WHO), burnout is classified and addressed as an “occupational phenomenon,” rather than a condition (Edú-Valsania et al., 2022), although it is one of the most discussed and researched mental health problems from today's society (Maslach and Leiter, 2016; Schaufeli and Buunk, 2003). By definition, the burnout syndrome is developed in response to the presence of prolonged stress associated with the workplace and is known to have three dimensions: emotional exhaustion, decreased job satisfaction and depersonalization (World Health Organization, 2019).

In academia, burnout can be a concern for everyone, from faculty members to students. Although the latter are not employed, the structured learning activities in which they participate can put as much pressure on them as a real job, and can often lead to exhaustion caused by study demands, cynicism about learning and a low self-efficacy in their academic work (Rahmati, 2015; Shadid et al., 2020). In the particular case of health sciences students, the overall prevalence of burnout is quite high, with reviews reporting figures ranging from 33%−55% (Almutairi et al., 2022; Frajerman et al., 2019). Romanian health science students are not spared from this risk, especially since they are exposed to sources of stress related to the particularities of the Romanian educational system, such as revoking the students' tax-free status, if their GPA is low, or the poor academic valorization of their performance (Diaconescu et al., 2024).

In terms of short-term consequences, burnout has been shown to have a negative impact on mental and physical health, with many students being at risk for anxiety, depression, insomnia and substance abuse (Walburg, 2014; Wing et al., 2018). There is also a long-term cost of burnout, as students who experience burnout during their academic studies are often prone to medical errors, affecting the quality of life and overall prognosis of their patients (Lee et al., 2020; McTaggart and Walker, 2022; Kumar, 2016).

When attempting to address burnout in academic health sciences settings, prevention has been shown to be far more effective than treatment (Shreffler et al., 2020; Williams et al., 2015; Pathipati and Cassel, 2018; Dyrbye et al., 2008). In this respect, one solid argument for prevention (e.g., for screening and early action against burnout) is made by the small percentage (26.8%) of students completely recovering from burnout, once it occurs (Bland et al., 2014). At the individual level, activities such as exercising, meditation, having a good sleep schedule, spending quality time with family and friends, limiting alcohol consumption, taking breaks and being well organized in learning have been reported to be effective (Merlo and Rippe, 2021; Yin et al., 2022; Popa-Velea et al., 2021). At the organizational level, action tools include for example adapting study programs, providing students with a steady workload (Alarcon et al., 2011; Aufricht et al., 2019), as well as providing on-campus counseling (Kilic et al., 2021). Additional organizational strategies may aim to create a work environment and culture in which mental illness is not stigmatized, provide psycho-educational seminars on burnout symptoms and information on available solutions, promote self-care practices and capitalize on digital solutions against burnout (Chetlen et al., 2019; Sultana et al., 2020).

As a general principle, to maximize their effectiveness, burnout prevention strategies should be adapted to both individual and organizational resources (Márquez, 2022). In this respect, additional factors that may have an additional contribution in their personalization, such as the socio-cultural background (age, gender), the specialty and the position in the study cycle (Grigorescu et al., 2020; Nituica et al., 2021; Ardekani et al., 2021) should be taken into account. In this complex landscape, the COVID-19 pandemic has recently brought new challenges to health sciences students, regarding the exposure to burnout and availability of prevention. Substantial changes in teaching (Goudarzi et al., 2024; Rose, 2020) required sudden adjustments in students' coping, while traditional strategies to prevent burnout, such as social support, may have been equally affected. In this regard, data from the literature indicate an association between social alienation perceived by many students during the pandemic and decreased instrumental support and interpersonal connectivity (Son et al., 2020; Restauri and Sheridan, 2020; Yin et al., 2021; Chen et al., 2023).

In the particular case of Romanian health sciences students, the COVID-19 pandemic has added a large amount of stress on the population and young people in general (isolation at home, avoiding physical contacts and meetings, the impossibility of spending free time with friends), but also on health sciences students (through specific circumstances, such as online learning, substantial changes in training, and high risk of exposure to infection). The Romanian educational system had to adapt quickly to these challenges, this representing an additional source of stress and burnout (Silistraru et al., 2022; Armean et al., 2021).

Following the COVID-19 pandemic, the burnout prevention strategies among health sciences students may have changed, which makes it necessary to investigate this issue and identify new tools for the development of educational policies. Specifically, this kind of information could not only contribute to filling a gap in the current literature, but also help to better target prevention resources in the academic environment, in the sense of increasing their benefit-cost ratio.

The aim of this study was to investigate, qualitatively and quantitatively, academic burnout, and the perception of effective prevention strategies among Romanian health sciences students.

## 2 Method

### 2.1 Study design

The design of the study was transversal and comprised two sections: (1) a quantitative analysis, evaluating burnout and its components, as well as the perceived efficiency of prevention strategies against burnout; (2) a survey investigating the self-reported preventive strategies against academic burnout.

### 2.2 Study participants

The participants were undergraduate students undergoing their training at the “Carol Davila” University of Medicine and Pharmacy Bucharest, Romania (CDUMP). This university is the largest medical school in Romania, gathering students from all regions of the country, thereby offering a relevant sample.

The inclusion criteria were: (a) age over 19 years old; (b) having the status of undergraduate students in the abovementioned institution; (c) giving the informed consent to participate in the study; and (d) completing all study instruments.

Exclusion criteria were represented by: (a) current self-reported somatic or psychiatric morbidity and (b) lack of completion of one or more of the study instruments. The study participants were selected through voluntary recruitment. Considering the highly- demanding schedule of health sciences students, this was considered as the most pragmatic and ethically justifiable method. Voluntary recruitment helped reduce response bias, as students who chose to take part in the study did so out of genuine interest rather than obligation. To ensure relevance of the study findings, established guidelines were used for minimum sample size estimation. The minimum required sample size was determined to be *n* = 274, considering a 90% confidence interval and a ±5% margin of error.

From the total of 311 students who agreed to participate, 6 students were eliminated for not having met the inclusion and exclusion criteria. Finally, 305 students participated in the study [36 (11.80%) men and 269 (88.20%) women]. Mean age was 21.90 (SD = 1.91).

The students came from the four faculties of CDUMP (General Medicine, Dental Medicine, Pharmacy, and Midwifery and Nursing). The vast majority of them came from General Medicine (82.30%) and from the third to sixth study years (the clinical track) (68.52%). A full description of their socio-demographic characteristics is depicted in Table 1.

**Table 1:** Socio-demographic characteristics of study participants.

**Variables**		**Statistics**				
**Quantitative**		***Mean***	**SD** ^*^	***Min***.	***Max***.	**SEM** ^†^
Age		21.9	1.91	19	26	0.11
*Qualitative*		*N*	%			
Gender	Male	36	11.80			
	Female	269	88.20			
Field of study	General Medicine	251	82.30			
	Pharmacy	30	9.83			
	Dental Medicine	17	5.57			
	Midwifery and Nursing	7	2.30			
Study year	First	43	14.10			
	Second	53	17.38			
	*Preclinical (Total)*	96	31.48			
	Third	59	19.34			
	Fourth	43	14.10			
	Fifth	35	11.48			
	Sixth	72	23.61			
	*Clinical (Total)*	209	68.52			

^*^SD, standard deviation; ^†^SEM, standard error of the mean.

### 2.3 Procedure

The study was run between November 2022 and March 2023. All students in the university were informed about the study and its objectives and invited to participate through organizational emails and social media university groups. The study protocol comprised:

- a quantitative analysis, using a series of psychometric instruments, to determine the objective and subjective levels of burnout, and the efficiency of individual and organizational prevention strategies;
- a survey, consisting in a series of open-ended questions focused on listing the strategies considered by students to be useful in preventing burnout.

Following expressing interest for the study theme, a convenience sample of self-selected participants was established. After applying the inclusion and the exclusion criteria, the final study sample was reached. Before participating in the research, the students were provided with explanations about the study and were asked if they wanted to participate in the study or not. Before answering, all students completed an informed consent form. The study was run in accordance with the World Medical Association Declaration of Helsinki and was approved by the CDUMP Ethics Committee (no. 15141/05.06.2022).

All questions were administered online and distributed through the social media groups of the university. A researcher (AM) was available by email, in case there were questions related to the process of filling the questionnaires. All responses were processed anonymously and a numerical code was assigned for each participant. The collected data were accessible exclusively to study researchers (AM, LD, OP-V, IS-B, and AIM). Regular didactic staff had no access to the distribution, collection or interpretation of questionnaires.

The interpretation of the questionnaires was performed independently by two researchers (LD, and OP-V) and cross-checked for congruence afterwards. Final results were included in a SPSS 21 (SPSS 144 Inc., Chicago, IL, USA) database.

The qualitative data analysis was focused on prevention methods of burnout perceived by the respondents as being efficient. It comprised raw content analysis, grouping in themes, and the thematic organization of the content (the detailed procedure is described in the “Data analysis” section below). The final results were also included in the study database.

### 2.4 Instruments

1. The Burnout Assessment Tool - general version (BAT) (Schaufeli et al., 2019) represents an instrument for diagnosing specific symptoms of burnout syndrome. It includes 23 items, grouped into four scales of core symptoms (“exhaustion,” “mental distance,” “cognitive impairment,” and “emotional impairment”) and which can be interpreted separately or together. An additional scale of secondary symptoms includes 10 items, grouped into two subscales (“psychological distress” and “psychosomatic symptoms”), which are interpreted as a whole. Response options are on a Likert scale from 1 to 5, where 1 = “never” and 5 = “always.” The BAT scale shows good psychometric properties, with Cronbach's alpha index value between 0.89 and 0.96 (Schaufeli et al., 2020). In this study we used the Romanian standardized version of the BAT (Oprea et al., 2021), validated on students (Popescu et al., 2023).

2. The subjective perception of the students regarding their level of burnout was measured by using a visual analog scale. The students were asked to self-assess their perceived level of burnout and code it on a scale from 0 (“not at all exhausted, no burnout”) to 10 (“extremely exhausted, very high level of burnout”).

3. A survey comprising:

- two questions with preformed answers, at which the students had to rate (from 1 = “not useful at all” to 4 = “very useful”), several individual and organizational strategies identified in previous research studies as being useful for burnout prevention. The individual preventive strategies included: physical activity, relaxation and meditation, extra-curricular and volunteering activities, cognitive-behavioral therapy, and establishing supportive relationships. The organizational strategies were: promoting social support, re-evaluation of the academic program, information sessions, mentoring programs, increased access to psychological assistance programs, and increased access to programs improving self-efficacy and resilience;

- two open-ended questions, aiming to identify students' opinions and preferences regarding the efficiency of burnout preventive strategies:

Question 1: “What do you consider to be the most effective measures to prevent burnout syndrome, at an individual level? Please give 3 examples in the descending order of their usefulness.”

Question 2: “What do you consider to be the most effective measures to prevent burnout syndrome at the organizational level? Please give 3 examples of how your educational institution could help you in this respect, in the descending order of their usefulness.”

In addition, the participants provided information about their age at the time of the testing, gender, study year, and field of study (General Medicine, Pharmacy, Dental Medicine, Midwifery, and Nursing).

### 2.5 Data analysis

The data was processed using Office Excel (Microsoft Corporation©, 2013) and IBM SPSS Statistics 26 (IBM Corporation©, 2019). Descriptive analysis (including mean, standard deviation, lowest/highest values, and standard error of the mean) were used to depict socio-demographic variables, burnout scores, and burnout prevention strategies.

The statistical analysis included independent samples *t-*tests, which were used to assess gender differences, differences based on study profile (preclinical vs. clinical years), and differences in individual prevention strategies based on burnout scores. Since the assumptions of normality were not met for all variables, Mann- Whitney U tests were applied to compare differences in organizational prevention strategies according to burnout scores.

Additionally, a multivariate analysis of variance (MANOVA) was conducted to examine differences in burnout levels and preventive strategies across respondents' study profiles. In this analysis, the dependent variables included burnout scores (assessed using the Burnout Assessment Tool), self-reported effectiveness of individual preventive strategies, and self-reported effectiveness of organizational prevention strategies. The independent variables consisted of gender, study profile (preclinical vs. clinical), and burnout severity.

Assumptions for each statistical test were checked prior to conducting the analyses. When normality assumptions were violated, appropriate non-parametric alternatives were used. A significance level (α) of 0.05 was set for all statistical tests, with *p*-values below this threshold considered statistically significant.

Thematic analysis was used for assessing the free answers given by the participants regarding the methods they considered to be the most effective in preventing burnout. The answers consisted in three words that they thought of, in response to the open-ended questions depicted in Section 2.4. All the steps specific to thematic analysis (Eldh et al., 2020; Nasa et al., 2021; Naeem et al., 2023) were followed. Microsoft Excel was used to organize and categorize the qualitative data. First, a literature search was run to gather information related to burnout prevention strategies in the academic context. Then the participants' responses were transcribed into an Excel table to identify recurring keywords and establish codes. Three researchers (AM, LD, and IS-B) independently performed the grouping of codes into themes, using Microsoft Excel, which allowed for systematic organization and categorization of the data. After this independent coding, the three researchers compared their theme groupings and participated in a discussion to address any differences in code classification or theme definitions. Inconsistencies in code classification or theme definition were deliberated and resolved. Finally, two other researchers (OP-V and AIM) reviewed the agreed-upon themes and the associated coded data, further analyzing and refining the themes. This additional review helped verify the consistency of the thematic structure and refine theme definitions where necessary. The researchers achieved consensus on a definitive collection of themes that reflect the data. The data were finally categorized into individual and organizational strategies. To ensure the reliability of the coding process, we assessed inter-rater reliability by calculating the percentage of agreement among the three researchers' initial theme groupings. This analysis showed a high level of agreement (80%), indicating consistent coding practices. Any remaining discrepancies were settled during the consensus meeting as described above.

## 3 Results

### 3.1 Socio-demographic variables

The distribution of the participants by age, gender, field of study, and study year is displayed in Table 1.

### 3.2 Burnout scores

The detailed burnout scores, including the scores of burnout sub-components are displayed in Table 2.

**Table 2:** Burnout scores.

**Characteristics**		**n**	**Mean**	SD^*^	**Min**.	**Max**.	**SEM^†^**
Diagnosed burnout (overall)		305	3.05	0.67	1.32	5.00	0.04
Core symptoms	Exhaustion	305	3.41	0.75	1.50	5.00	0.04
	Mental distance	305	2.44	0.83	1.00	5.00	0.05
	Cognitive impairment	305	2.99	0.92	1.00	5.00	0.05
	Emotional impairment	305	3.03	0.89	1.00	5.00	0.05
Secondary symptoms	Overall	305	3.03	0.76	1.30	4.90	0.04
	Psychological distress	305	3.37	0.86	1.20	5.00	0.05
	Psychosomatic symptoms	305	2.69	0.86	1.00	5.00	0.05
Self-reported burnout		305	5.71	2.25	0.00	10.00	0.13

^*^SD, standard deviation; ^†^SEM, standard error of the mean.

Burnout scores were above average (including all its sub-components), with highest scores in exhaustion and psychological distress, and lowest in mental distance and psychosomatic symptoms. The burnout self-reported scores were consistent with those obtained through psychometric evaluation.

### 3.3 Prevention strategies

The effectiveness of burnout prevention strategies is displayed in Table 3.

**Table 3:** Prevention strategies.

**Characteristics**		**n**	**Mean**	SD^*^	**Min**.	**Max**.	**SEM^†^**
Individual	Physical activity	305	3.36	0.80	1.00	4.00	0.05
	Relaxation and meditation techniques	305	3.32	0.87	1.00	4.00	0.05
	Extracurricular and volunteering activities	305	2.70	0.99	1.00	4.00	0.06
	Cognitive- behavioral therapy	305	3.36	0.85	1.00	4.00	0.05
	Establishing supportive relationships	305	3.75	0.55	1.00	4.00	0.03
Organiza- tional	Promoting social support	305	3.51	0.72	1.00	4.00	0.04
	Re-evaluation of the academic program	305	3.84	0.48	1.00	4.00	0.03
	Information sessions	305	3.39	0.83	1.00	4.00	0.05
	Mentoring programs	305	3.38	0.79	1.00	4.00	0.05
	Increasing access to psychological assistance programs	305	3.72	0.58	1.00	4.00	0.03
	Programs that increase self-efficacy and resilience	305	3.65	0.62	1.00	4.00	0.04

^*^SD, standard deviation; ^†^SEM, standard error of the mean.

At the individual level, burnout prevention was assessed as being the most effective through supportive relationships and the least effective through extracurricular and volunteering activities.

Regarding burnout prevention at the organizational level, the re-evaluation of the academic program was considered by students to be the most effective, while information sessions were assessed as being the least effective.

### 3.4 Gender differences

They are described in Table 4, which comprises two sections: symptoms and prevention strategies (individual and organizational).

**Table 4:** Gender differences regarding burnout and prevention strategies against burnout.

**Characteristics**	**Gender**		**df**	**t**	**p**	**Mean difference 95% CI**	**Cohen's d**
**A. Burnout symptoms**	**Men** ^*^	**Women** ^*^					
Diagnosed burnout	2.73	3.10	303	3.15	0.002	0.37 [0.14, 0.60]	0.56
Exhaustion	2.99	3.46	303	3.62	0.001	0.47 [0.21, 0.73]	0.63
Mental distance	2.39	2.45	303	0.42	0.675	0.06 [−0.21, 0.34]	0.08
Cognitive impairment	2.72	3.02	303	1.88	0.061	0.30 [−0.01, 0.62]	0.40
Emotional impairment	2.58	3.09	303	3.34	0.001	0.52 [0.21, 0.82]	0.68
Secondary symptoms	2.64	3.08	303	3.27	0.001	0.44 [0.17, 0.70]	0.64
- Psychological distress	2.97	3.42	303	2.94	0.004	0.45 [0.15, 0.74]	0.60
- Psychosomatic symptoms	2.31	2.74	303	2.40	0.021	0.42 [0.13, 0.72]	0.60
Self-reported burnout	4.94	5.81	303	2.19	0.030	0.87 [0.09, 1.65]	0.40
**B. Burnout prevention strategies**							
B.1. Individual	Men^*^	Women^*^	df	t	p	Mean difference 95% CI	Cohen's d
Physical activity	3.25	3.38	303	0.91	0.361	0.13 [−0.15, 0.41]	0.16
Relaxation and meditation techniques	3.03	3.36	303	2.19	0.029	0.33 [0.03, 0.63]	0.39
Extracurricular and volunteering activities	2.75	2.70	303	−0.31	0.755	−0.05 [−0.37, 0.27]	0.06
Cognitive-behavioral therapy	3.17	3.38	303	1.44	0.152	0.21 [−0.08, 0.50]	0.25
Establishing supportive relationships	3.75	3.75	303	0.009	0.992	0.01 [−0.26, 0.26]	0.01
B.2. Organizational	Men^**^	Wo-men^**^	Mann- Whitney U	Z	p	Effect size r for Mann Whitney U	
Promoting social support	139.93	154.75	4,371.50	−1.11	0.269	0.06	
Re-evaluation of the academic program	147.89	153.68	4,658.00	−0.63	0.527	0.04	
Information sessions	137.57	155.07	4,286.50	−1.26	0.207	0.07	
Mentoring programs	127.99	156.35	3,941.50	−2.02	0.043	0.12	
Increasing access to psychological assistance programs	133.56	155.60	4,142.00	−1.96	0.049	0.11	
Programs that increase self-efficacy and resilience	139.43	154.82	4,353.50	−1.25	0.212	0.07	

^*^Mean score; ^**^Rank mean.

Regarding individual strategies against burnout, women reported higher levels of effectiveness for relaxation and meditation techniques, physical activity and use of cognitive-behavioral therapy than men, but only the first difference was statistically significant. In what concerned the methods of prevention at the organizational level, women supported, significantly higher than men, the efficacy of mentoring programs and—at the limit of statistical significance—the increased access to psychological assistance programs.

### 3.5 Differences regarding burnout and burnout prevention, by study profile

The multivariate analysis of variance analysis (MANOVA) revealed that there were no statistically significant differences between the respondents' study profile in terms of diagnosed burnout and self-reported burnout. However, in terms of burnout prevention strategies, there were significant differences between study profiles, in what concerned:

- relaxation and meditation techniques (F = 4.47, df = 3, *p* < 0.004; Cohen's d = 0.88; Eta-squared = 0.04) (highest mean score at Dental Medicine: 3.73, lowest mean score at General Medicine: 3.24);
- promoting social support (F = 2.90, df = 3, *p* < 0.035; Cohen's d = 0.69; Eta-squared = 0.03) (highest mean score at Dental Medicine: 3.87; lowest mean score at Midwifery and Nursing: 3.43).

### 3.6 Differences regarding burnout and burnout prevention, by study track (preclinical/clinical)

No statistically significant differences were identified between preclinical and clinical study domain, in what concerned diagnosed burnout, self-reported burnout, and effectiveness of burnout prevention strategies (both at the individual and the organizational level).

### 3.7 The association between self-reported burnout and perceived effectiveness of prevention strategies

We tested this relationship, after splitting the participants in quartiles, according to their self-reported subjective burnout. The comparisons were realized between Q1 (low self-reported burnout, < 4.00) and Q4 (high self-reported burnout, >7.00) (Table 5).

**Table 5:** Perceived effectiveness of prevention strategies, by self-reported burnout.

**Preferred prevention strategies**		**Q1 (*****n*** = **61)**		**Q4 (*****n*** = **66)**		**df**	**t**	**p**	**Cohen's d**
		**Mean**	**SD** ^*^	**Mean**	**SD** ^*^				
Individual	Physical activity	3.64	0.63	3.23	0.82	121.31	3.19	0.002	0.56
	Relaxation and meditation techniques	3.52	0.65	3.30	0.86	120.31	1.65	0.102	0.29
	Extracurricular and volunteering activities	2.92	1.04	2.61	0.98	125	1.75	0.105	0.31
	Cognitive-behavioral therapy	3.16	0.88	3.47	0.79	125	−2.07	0.041	0.37
	Establishing supportive relationships	3.90	0.30	3.74	0.62	95.90	1.87	0.064	0.33
Organizational	Promoting social support	3.49	0.74	3.67	0.59	114.38	−1.46	0.147	0.27
	Re-evaluation of the academic program	3.67	0.57	3.92	0.32	92.71	−3.05	0.003	0.55
	Information sessions	3.36	0.90	3.53	0.75	117.39	−1.15	0.251	0.21
	Mentoring programs	3.36	0.73	3.44	0.79	125	−0.58	0.561	0.11
	Increasing access to psychological assistance programs	3.61	0.67	3.80	0.40	96.93	−2.00	0.049	0.35
	Programs that increase self-efficacy and resilience	3.59	0.67	3.76	0.56	117.17	−1.53	0.129	0.28

^*^SD, standard deviation.

In students with low self-reported burnout, the individual prevention methods considered the most effective were, in descending order, establishing supportive relationships, physical activity and relaxation and meditation techniques, while in students with high self-reported burnout, this hierarchy was slightly changed, with the introduction of cognitive-behavioral therapy on the second place of preferred strategies.

In what concerns the organizational strategies, all students, irrespective of their burnout, situated at the top of the list the re-evaluation of the academic program, the increased access to psychological assistance programs and the programs increasing self-efficacy and resilience.

Despite these commonalities, significant differences were identified at the quantitative level between students with low and high self-reported burnout. These differences concerned physical activity, cognitive-behavioral therapy, re-evaluation of the academic program, and access to psychological assistance programs. Specifically, students with lower self-reported burnout significantly valued physical activity higher and the other three prevention methods lower.

### 3.8 The association between diagnosed burnout and perceived effectiveness of prevention strategies

This relationship was evaluated after splitting the participants in quartiles, according to their diagnosed burnout, measured through their BAT score. The comparisons were realized between Q1 (low burnout, score < 2.61) and Q4 (high burnout; score > 3.50) (Table 6).

**Table 6:** Perceived effectiveness of prevention strategies, by diagnosed burnout.

**Preferred prevention strategies**		**Q1 (n** = **76)**		**Q4 (n** = **84)**		**df**	**t**	**p**	**Cohen's d**
		**Mean**	**SD**	**Mean**	**SD**				
Individual	Physical activity	3.64	0.61	3.24	0.82	152.26	3.60	0.001	0.55
	Relaxation and meditation techniques	3.47	0.68	3.35	0.81	158	1.08	0.284	0.16
	Extracurricular and volunteering activities	2.97	0.99	2.51	0.98	158	2.97	0.003	0.47
	Cognitive-behavioral therapy	3.17	0.87	3.50	0.77	158	−2.54	0.012	−0.40
	Establishing supportive relationships	3.89	0.35	3.73	0.63	132.41	2.12	0.036	0.31
Organizational	Promoting social support	3.43	0.75	3.49	0.77	158	−0.45	0.656	−0.08
	Re-evaluation of the academic program	3.76	0.49	3.89	0.44	151.87	−1.76	0.080	−0.28
	Information sessions	3.33	0.92	3.48	0.77	147.09	−1.10	0.275	−0.18
	Mentoring programs	3.32	0.80	3.52	0.70	158	−1.75	0.083	−0.27
	Increasing access to psychological assistance programs	3.59	0.677	3.86	0.39	116.28	−3.00	0.003	−0.50
	Programs that increase self-efficacy and resilience	3.61	0.634	3.74	0.56	150.73	−1.34	0.165	−0.22

At the individual level, students with low burnout scores considered effective the establishment of supportive relationships, followed by physical activity and relaxation-meditation techniques. Despite conserving the same assessment for the first and the third strategy above, students with high burnout scores designated cognitive-behavioral therapy as the second most useful burnout prevention strategy.

In terms of organizational strategies, there was a consensus among all students, irrespective of their burnout scores, regarding the three most effective preventive strategies, namely re-evaluation of the academic program, increasing access to psychological assistance programs and to programs that increase self-efficacy and resilience.

The *t*-test for independent samples revealed quantitative differences between students with low and high burnout scores regarding physical activity and extracurricular and volunteering activities (mentioned by those in the low burnout group) vs. cognitive-behavioral therapy and increased access to psychological assistance programs (mentioned by those in the high burnout group).

### 3.9 Freely chosen burnout preventive strategies

A thematic analysis was conducted to explore the individual strategies freely chosen by study participants to prevent burnout (Table 7). The responses revealed a diverse range of coping mechanisms that were categorized into individual and organizational strategies. A recurring theme was the need to establish clear boundaries between work and personal life. Participants highlighted the significance of setting realistic work goals, taking breaks, and engaging in leisure activities to prevent burnout.

**Table 7:** Freely chosen burnout preventive strategies.

**Theme**	**Specific activities**	%^*^
**A. Individual strategies**		
Detachment	Outdoor activities, relaxation, recreation, taking breaks, hobbies, traveling	34.49
	Time with friends, family	8.46
	Religion, faith	0.33
	Disconnection from the online environment	0.22
	*Total*	*43.50*
Active actions related to academic stress	Organization, setting goals, objectives and limits, learned responsibility	20.50
	Stress management and positive thinking	3.25
	Self-focus	1.63
	Managing the academic environment	1.30
	*Total*	*26.68*
Healthy lifestyle	Sleep, optimal rest	11.50
	Sport, physical activities	8.79
	Balanced diet, sufficient hydration	4.77
	*Total*	*25.06*
Specialized help	Cognitive-behavioral therapy, counseling	4.77
	*Total*	*4.77*
**B. Organizational strategies**		
Academic reorganization	More structured materials and improving the quality of the courses, optimal training of teaching staff	11.37
	Optimal organization of evaluation methods	9.74
	Re-evaluation of the academic program	4.76
	Greater emphasis on practical activities	2.90
	*Total*	*28.77*
Program optimization	Revision of the timetable and better organization	21.23
	Breaks, holidays	4.18
	*Total*	*25.41*
Active interventions of the institution	Promoting mental health and providing therapy services	12.88
	Organization of courses and information campaigns about burnout, courses for stress and time management	4.87
	*Total*	*17.75*
Promoting an environment conducive to intellectual development	Communication and interest in students' opinions, changing the attitude of teachers and faculty	8.47
	Decrease in workload, more balanced tasks	2.78
	Providing a conducive environment for study and for students' needs	1.86
	Motivating students and appreciating their results	1.39
	*Total*	*14.50*
Promoting social support among students	Reduction of competitiveness among students and academic pressure	6.15
	Organization of events for students (within or outside the university environment)	5.92
	Promoting peer support (support groups, study groups	1.51
	*Total*	*13.58*

^*^For each of the two categories, A and B, the percentages represent the frequency of each reported prevention method across all received responses.

## 4 Discussion

This study aimed to investigate academic burnout and the perception of effective prevention strategies against it in Romanian health sciences students.

### 4.1 Burnout scores

Overall, burnout scores were higher than average, for both self-reported burnout and diagnosed burnout. On the subcomponents, the highest scores were obtained for exhaustion and psychological distress. Although the scores for psychosomatic symptoms were reported to be low, the association between psychological and physical symptoms (distress and exhaustion, respectively) poses a risk, potentially creating the premise of a vicious cycle mechanism where these symptoms reinforce each other. A positive aspect is represented by the consistency between self-reported burnout and diagnosed burnout calculated by the BAT instrument, suggesting a good awareness of the participants regarding their problems derived from academic distress. Gender differences were quite evident, with women displaying higher levels of burnout than men, in terms of general burnout (especially exhaustion), emotional impairment, and secondary symptoms. This is consistent with data from literature, which reported a greater sensitivity of women to distress, including to academic pressures, and manifested as emotional imbalance and exhaustion (Fiorilli et al., 2022; Fares et al., 2016; Vidhukumar and Hamza, 2020). A possible explanation for this is also their greater involvement in learning tasks, with more prolonged effort, all potentially leading to burnout and its externalization as secondary symptoms. In practical terms, this finding once again suggests the need for more substantial efforts in detecting and managing academic burnout in female students, especially in the early stages of their academic training.

### 4.2 Burnout prevention strategies

At the individual level, participants found supportive relationships to be the most effective in preventing burnout. This highly perceived effectiveness is probably due to its immediate impact on important personal aspects, such as the feeling of self-fulfillment, the maintenance of one's social identity (Hirsch, 1986), or a better coping with stressors (Schwarzer and Knoll, 2007). These mechanisms proved to be especially important during and after the COVID-19 pandemic, considering the higher need of cohesion, collaborative relationships and solidarity in the academic environments, especially in healthcare-related ones. Also, participants found the use of relaxation-meditation as helpful strategies, regardless of their self- reported or diagnosed burnout.

In terms of differences, participants with high burnout scores considered cognitive-behavioral therapy as a reliable tool, while those with low burnout scores replaced it with physical activity. An explanation for this could be the perception of burnout symptoms in the high burnout students group as reflecting a significant deterioration of their previous mental status (thereby requiring specialized care), while, oppositely, in the low burnout students' group, this could be labeled as a temporary problem, which could be addressed more simply, through physical exercise. Equally, the high burnout group may have already tried physical exercise, without significant effects. Finally, despite the fact that physical exercise can have a significant indirect effect on academic burnout via self-efficacy and resilience (Chen et al., 2022), high burnout students could perceive this effect as insufficient or absent, as they already display significantly low levels of these characteristics, thereby orienting themselves toward psychotherapy. These results could be useful in regulating the intervention/prevention balance and should be considered when designing burnout prevention programs in the academic environment.

At the organizational level, regardless of their burnout scores and how they were measured (by self- report or BAT), participants perceived academic overload as the main contributor to burnout. In this context, re-evaluating the university program (in the sense of reducing the volume of studies and ensuring a balanced schedule) can be useful both for prevention and for intervention in the case of burnout. This can be complemented by increasing access to psychological support programs and programs that increase self-efficacy and resilience. In a nursing profession that involves intense study, long-term effort, and constant confrontation with human suffering, students identify these two strategies as important for improving coping skills and resistance to burnout. In our opinion, the near-unanimous emphasis on these anti-burnout strategies most likely reflects a lack of provision and/or effectiveness of these elements in health sciences academia. This is, moreover, one of the reasons why health sciences continue to be perceived as a long and difficult road, which essentially involves overwhelmingly a solitary effort, with minimal or absent organizational support (Choy and Wong, 2017; Vogel, 2018; Ofei-Dodoo et al., 2021). In particular, information sessions on burnout were considered the least effective, essentially exposing current organizational support in this area as not practice-oriented (i.e., instrumental support), but rather aimed at providing purely theoretical knowledge.

Two specific variables appear to influence perceptions of burnout prevention strategies:

- gender: at the individual level, women relied, significantly more than men, on relaxation and meditation, this potentially reflecting their subjective benefit, described in the literature as greater from these techniques (Rojiani et al., 2017). At the organizational level, women were more open to mentoring and psychological assistance programs. This is consistent with the high importance given to mentoring in the health sciences field, as it can provide real emotional support and counseling (Nimmons et al., 2019). Previous studies have shown that female students value mentoring as a key contribution to their personal development (Cross et al., 2019), particularly regarding same-gender role models in specific areas of Medicine (Bettis et al., 2019). In this regard, current data from the literature states that women are dissatisfied with the number of mentoring opportunities available to them (Bhatnagar et al., 2020);
- study profile: relaxation and meditation were more appreciated by students in Dental Medicine compared to General Medicine. This may reflect future dentists' greater familiarity with brief relaxation techniques and their rapid positive effects on patients, particularly in reducing anxiety (Lahmann et al., 2008; Alhazmi et al., 2022). Because anxiety is often associated to burnout (Koutsimani et al., 2019), they may identify relaxation, by extension, as helpful in their own burnout prevention. In contrast, for many of students in General Medicine, relaxation and meditation are often perceived as complementary procedures, and not interventions *per se*.

The free choice of burnout prevention strategies essentially provided a hierarchy of preferences, which at the individual level included, in descending order, detachment, active actions, healthy lifestyle, and specialized help. The most frequently invoked strategies included disconnecting, relaxing, and spending time with friends and family. Notably, specialist help was spontaneously mentioned in only 4.77% of the responses collected (mostly by participants with high burnout), potentially reflecting skepticism about its effectiveness and/or availability. The qualitative responses suggested that self-care techniques such as physical activity, eating a healthy diet, and practicing mindfulness are commonly considered as helpful stress-reduction strategies. Many participants underlined the importance of implementing relaxation techniques (such as meditation and breathing exercises) into their daily routines, which supports prior research on the function of mindfulness in burnout prevention. At the organizational level, the methods considered the most effective were academic reorganization and program optimization, while the least effective were the promotion of an environment conducive to intellectual development and the promotion of social support among students. It is possible that the last two directions were considered, compared to others, too vague and/or difficult to implement in practice.

In terms of the correlation between qualitative and quantitative analyses, the freely chosen preventive strategies were consistent with the quantitative results, pointing out that students with lower burnout levels (Q1) rated physical exercise higher, but those with greater burnout scores (Q4) preferred CBT. More specifically, participants who spontaneously indicated “exercise,” “sports,” or “going to the gym” as efficient prevention strategies had lower burnout levels. In contrast, those listed “therapy” or “professional counseling” as one of their top three options, frequently were classified as very burned out through quantitative analysis. This consistency supports the idea that lower burnout symptoms can be managed by lifestyle changes, whereas more severe symptoms prompt students to seek professional assistance.

As a whole, our study supports the idea that burnout in health sciences students can reach significant levels (Almutairi et al., 2022; Frajerman et al., 2019) and have short- and long-term consequences, affecting mental wellbeing and influencing academic and professional achievements (Madigan and Curran, 2021). Also, our findings are consistent with the conclusions of a recent meta-analysis (Madigan et al., 2024), which identified most important interventions that have proven to be effective in preventing burnout in students (mindfulness-based stress reduction, rational emotive behavior therapy, psychoeducation, cognitive-behavioral therapy, and exercise).

In terms of methodology, our study combined quantitative and qualitative research, thereby providing a complex picture of the personal and institutional factors that may mitigate academic burnout. Our findings emphasize the necessity of a multimodal strategy for burnout prevention that includes both personal coping methods and institutional support mechanisms. Universities should explore implementing focused interventions that promote self-care routines, improve time management skills, and provide mental health resources tailored to the unique challenges that health sciences students face.

### 4.3 Limitations

The results from our sample may have limited generalizability to all students, brought by the recruitment method and by the fact that the data were collected online and were self-reported. We relied mainly on the convenience method of sampling and on the students' self-selection through access to the organizational email of the university. Students exhibiting greater online activity or a specific interest in burnout related subjects may have been more motivated to participate. We attempted to lower the derived bias risks, by allowing ample time for participation and by ensuring voluntary participation. Another study limitation is the skewed gender ratio (with a significantly higher number of female participants). Still, one may note that other studies focused on academic burnout also report a skewness of the study sample in terms of gender, this being common in Romanian universities with a humanistic profile [e.g., male/female ratio in Psychology = 1: 6.57 (Popescu et al., 2023)].

For future research, better sampling methods could be stratified sampling (which could eliminate the skewed gender ratio) or cluster sampling (which would ensure a more accurate representation of students both in terms of year of study and study profile).

Another limitation is represented by the cross-sectional nature of this research, which precludes claims of causality. The study is a snapshot of a situation at a given moment, both in terms of the level of burnout symptoms in health sciences students, and in terms of their strategies to prevent burnout, in the aftermath of the COVID-19 pandemic. Future research could take into consideration a longitudinal design, through which burnout symptoms can be observed throughout the whole study cycle. This could equally follow the efficiency of prevention strategies against burnout, especially the organizational ones.

Finally, we did not take into account additional elements (e.g., personality traits, curriculum overload, and cultural traits) that could influence the prevalence of burnout. This could have provided valuable information and strengthen the study's conclusions. Future studies could include these variables and investigate burnout prevention measures in a larger variety of cultural and educational contexts.

## 5 Conclusions

The results of the current study reveal that indicators of burnout syndrome are largely experienced by health sciences students. Women are more likely, when experiencing burnout, to associate emotional impairment and secondary symptoms. Supportive relationships, followed by relaxation and meditation are considered helpful preventive strategies, while cognitive-behavioral therapy is considered especially effective to prevent burnout in students with higher burnout scores. A series of other prevention strategies, such as detachment, proactive actions, healthy lifestyle, flexibility of the university curriculum and access to programs that provide support and increase self-efficacy and resilience, were independently considered by the respondents, as potentially useful.

These results, but also previous research in this area run in our organization and five other European countries (https://bendit-eu.eu/policy-toolkit/BENDit-EU%20policy%20toolkit%20.pdf) suggest a series of concrete measures that could prove to be effective in the prevention and/or early management of student burnout. They include available tools for individuals to self-assess burnout; regular screening for distress; empowerment and education of trainees to prioritize their own health; revision of curricular time and work assignments to allow for time-off during a typical workday; assessment of perceived and feared discrimination; adequate access to conveniently located mental health counselors; implementing a resilience curriculum; use of smaller learning communities to build group cohesion and social support; monitoring and offering support to trainees, in case of major life events; organization of social activities to foster peer–peer and peer–faculty relationships; access to fitness facilities; encourage faculty staff development to raise awareness and facilitate a positive learning environment. Implementation of these measures could be very useful in designing sustainable and effective pathways to address burnout in health sciences academic settings.

## Data Availability

The raw data supporting the conclusions of this article will be made available by the authors, without undue reservation.
